# Regulation of the Expression of DAPK1 by SUMO Pathway

**DOI:** 10.3390/biom9040151

**Published:** 2019-04-17

**Authors:** Qingshui Wang, Xiuli Zhang, Ling Chen, Shuyun Weng, Yun Xia, Yan Ye, Ke Li, Ziqiang Liao, Pengchen Chen, Khaldoon Alsamman, Chen Meng, Craig Stevens, Ted R. Hupp, Yao Lin

**Affiliations:** 1 Provincial University Key Laboratory of Cellular Stress Response and Metabolic Regulation, College of Life Sciences, Fujian Normal University, Fuzhou 350117, China; wangqingshui@fjnu.edu.cn (Q.W.); 15980239296@163.com (X.Z.); chenling654321@163.com (L.C.); wsy09080700@163.com (S.W.); xiayunnyyl@163.com (Y.X.); m18759141945@163.com (Y.Y.); 13107673087@163.com (K.L.); liaoziqiangcontact@163.com (Z.L.); yb77620@umac.mo (P.C.); mmenger@126.com (C.M.); 2 Department of Clinical Laboratory Sciences, College of Applied Medical Sciences, Imam Abdulrahman bin Faisal University, Dammam 34212, Saudi Arabia; kmalsamman@iau.edu.sa; 3 School of Applied Sciences, Edinburgh Napier University, Edinburgh EH11 4BN, UK; C.Stevens@napier.ac.uk; 4 Institute of Genetics and Molecular Medicine, Cell Signaling Unit, CRUK p53 Transduction Group, University of Edinburgh, EH4 2XR EH4 2XR, UK; ted.hupp@ed.ac.uk

**Keywords:** DAPK1, SUMO, SENP, protein degradation, post-translational modification

## Abstract

Death Associated Protein Kinase 1 (DAPK1) is an important signaling kinase mediating the biological effect of multiple natural biomolecules such as IFN-γ, TNF-α, curcumin, etc. DAPK1 is degraded through both ubiquitin-proteasomal and lysosomal degradation pathways. To investigate the crosstalk between these two DAPK1 degradation pathways, we carried out a screen using a set of ubiquitin E2 siRNAs at the presence of Tuberous Sclerous 2 (TSC2) and identified that the small ubiquitin-like molecule (SUMO) pathway is able to regulate the protein levels of DAPK1. Inhibition of the SUMO pathway enhanced DAPK1 protein levels and the minimum domain of DAPK1 protein required for this regulation is the kinase domain, suggesting that the SUMO pathway regulates DAPK1 protein levels independent of TSC2. Suppression of the SUMO pathway did not enhance DAPK1 protein stability. In addition, mutation of the potential SUMO conjugation sites on DAPK1 kinase domain did not alter its protein stability or response to SUMO pathway inhibition. These data suggested that the SUMO pathway does not regulate DAPK1 protein degradation. The exact molecular mechanism underlying this regulation is yet to be discovered.

## 1. Introduction

Death-Associated Protein Kinase 1 (DAPK1) is an important serine/threonine kinase that is involved in multiple cellular processes such as apoptosis, autophagy, inflammation [1]. DAPK1 Plays a vital role in the anti-carcinogenic effects of many natural-based biomolecules, such as IFN-γ, TNF-α, curcumin, etc. [2,3]. Decreased expression of DAPK1 has been proven to be an unfavorable prognostic factor in bladder cancer, liver cancer and non-small cell lung cancer, etc. [4,5].

DAPK1 protein is composed of multiple functional domains including a catalytic kinase domain, a Ca2+/CaM auto-regulatory domain, eight ankyrin repeats, a Ras of Complex proteins (ROC)-C-terminus of ROC (COR) domain, a death domain and a serine-rich tail [6]. Dysregulation of DAPK1 expression or activity is often related to multiple diseases including cancer and stroke [7]. Recently, DAPK1 was also found to inhibit Hypoxia-inducible factor 1α (HIF-1α) in T cells [8], maintain epidermal tissue integrity through regulation of the microtubule cytoskeleton in C.elegans [9], and mediate pegylated Interferon-α (IFN-α)-induced suppression of hepatitis C virus (HCV) replication [10]. 

Expression of DAPK1 is often lost in cancers due to DNA methylation of the DAPK1 gene [11]. Due to DNA methylation, the expression of DAPK1 is lost in primary tumor samples of 26% of rectal cancer patients. Similarly, different degrees of DNA methylation of DAPK1 have also been found in lung cancer, leukemia, breast cancer, uterine cancer and prostate cancer [12,13,14]. Previous studies revealed that the expression of the DAPK1 protein does not match its expression of mRNA in some cancers, indicating that the regulation of DAPK1 expression is a complex process. The degradation of DAPK1 protein is controlled by both proteasomal and lysosomal degradation pathways [15]. Three ubiquitin E3s, Mind Bomb 1(Mib1) [16], C-terminus of Hsc70-interacting protein (CHIP) [17] and KLHL20-Cullin3-RBX1 complex [18], target DAPK1 for ubiquitin-proteasome system (UPS)-mediated degradation. The lysosomal degradation pathway of DAPK was found to be late compared to proteasome degradation. Proteins known to be involved in the DAPK1 protein of lysosomal degradation include s-DAPK, Tuberin (TSC2) and cathepsin B. The TSC complex, formed by two proteins (TSC1 (hamartin) and TSC2), is a major regulator of the mTORC1 signaling pathway [19]. In our previous work, we discovered that TSC2 and a splice variant of DAPK1 (s-DAPK1) induced the lysosomal degradation of DAPK1 [20,21]. Moreover, a lysosomal protease, cathepsin B, is able to cleave DAPK1 in response to Tumor Necrosis Factor Receptor 1 (TNFR-1) over-expression [22]. 

Growing evidence demonstrates that there is intense crosstalk between UPS and lysosome [23]. Ubiquitination on proteins such as p62 can lead to their degradation by both UPS and lysosome [23]. Although DAPK1 has been shown to be degraded by both degradation pathways, it is not clear whether the ubiquitin related signaling pathways contribute to its lysosomal degradation. To investigate the crosstalk between these two DAPK1 degradation pathways, we carried out a screen using a set of ubiquitin E2 siRNAs and identified that the small ubiquitin like molecule (SUMO) pathway regulates DAPK1 protein levels. 

SUMO is a family of ubiquitin-related modifiers that can be post-translationally conjugated to various substrates [24]. Intracellular proteins can be modified by SUMO, which affects substrate protein localization, stability, protein modification, and protein-protein interactions [25]. Five different SUMO paralogues have been reported in vertebrates, named SUMO-1 to SUMO-5 [24]. SUMO-1 shares 45% homology with SUMO-2/3, and there are only two amino acids difference between SUMO-2 and SUMO-3 [26]. SUMO-4 encodes a 95-amino acid protein having an 86% amino acid homology with SUMO-2 [27]. SUMO5 is a novel SUMO variant and contains a protein-coding sequence of 306 nucleotides [28]. The covalent modification reaction of SUMO is catalyzed by a series of enzymes including E1 activating enzyme (SAE1/SAE2), E2 binding enzyme (UBC9) and E3 ligase enzyme [29]. The process of SUMOylation is dynamic and reversible. A family of SUMO specific proteases (SENPs) are capable of removing SUMO from attached substrates and responsible for SUMO maturation [24]. Family members of SENPs include SENP-1, SENP-2, SENP-3, SENP-5, SENP-6 and SENP-7. The SENP family can be divided into three subfamilies based on the degree of amino acid sequence homology, cell localization, and substrate preference. The first subfamily comprises SENP-1 and SENP-2, which are located on the nuclear membrane and have the widest selection of substrates, which participate in the deubiquitination of proteins modified by SUMO-1 and SUMO-2/3 [30,31]. The second subfamily is SENP-3 and SENP-5. They are mainly found in nucleoli and involved in the synthesis of ribosomes and the regulation of cell mitosis [32]. The third subfamily is SENP-6 and SENP-7. They are located in the nucleus and are essential for the formation of multi-cluster ubiquitin chains [33,34].

In summary, the degradation of DAPK protein is regulated by both proteasome and lysosome. The research of E2 on degradation of DAPK1 protein will help to better understand the regulation of DAPK protein degradation by ubiquitin and ubiquitin-like small molecule signaling pathways and to discover the link between ubiquitin-related small molecule and lysosomal degradation signaling pathways.

## 2. Materials and Methods

### 2.1. Cell Culture and Transfection

HEK293 (human embryonic kidney cell line) and HCT116 (Human colon carcinoma) cells were obtained from ATCC (Manassas, MD, USA). Cell lines were examined for mycoplasma contamination using Mycoplasma Detection Kit (Vazyme, Nanjing, Jiangsu, China). Both cells were cultured in DMEM medium (Invitrogen, Carlsbad, CA, USA) supplemented with 10% fetal bovine serum (FBS) and a penicillin and streptomycin mixture at 37 °C with 5% CO_2_ in a humidified atmosphere. Before harvesting, cells were first washed twice with PBS and then scraped into 1 mL of PBS. PCDNA3-HA-DAPK1 was a gift from Ted R. Hupp (University of Edinburgh). Flag-SENP1 (Plasmid #17357, deposited by Edward Yeh), FLAG-SENP2 (Plasmid #18047, deposited by Edward Yeh), RGS-SENP3 (Plasmid #18048, deposited by Edward Yeh), RGS-SENP5 (Plasmid #18053, deposited by Edward Yeh), FLAG-SENP6 (Plasmid #18065, deposited by Edward Yeh), 3xFLAG-SENP7 (Plasmid #42886, deposited by Edward Yeh) and Flag TSC2 wt (Plasmid #12132, deposited by Cheryl Walker) were obtained from Addgene (Cambridge, UK). The DAPK1 mutant constructs were generated using the QuikChange Lightning Site-Directed Mutagenesis Kit from Vazyme (Nanjing, China). All plasmids were sequenced to verify the integrity of the constructs. UBE2A siRNA (E-009424-00-0005), UBE2B siRNA(E-009930-00-0005), UBE2C siRNA(E-004693-00-0005), UBE2D1 siRNA (E-009387-00-0005), UBE2D2 siRNA (E-010383-00-0005), UBE2D3 siRNA (E-008478-00-0005), UBE2E1 siRNA(E-008850-00-0005), UBE2E2 siRNA (E-031782-00-0005), UBE2E3 siRNA (E-008845-00-0005), UBE2G1 siRNA (E-010154-00-0005), UBE2G2 siRNA (E-009095-00-0005), UBE2H siRNA (E-009134-00-0005), UBE2I siRNA (E-004910-00-0005), UBE2J1 siRNA (E-007266-00-0005), UBE2J2 siRNA (E-008614-00-0005), UBE2L3 siRNA (E-010384-00-0005), UBE2M siRNA (E-004348-00-0005), UBE2N siRNA (E-003920-00-0005), UBE2NL siRNA (E-031625-00-0005), UBE2Q1 siRNA (E-008631-00-0005), UBE2R2 siRNA (E-009700-00-0005), UBE2S siRNA (E-009707-00-0005) and UBE2V2 siRNA (E-008823-00-0005) were purchased from Dharmacon (Lafayette, MA, USA). The transfection was performed using lipofectamine 2000 (Invitrogen, Carlsbad, CA, USA) according to the manufacturer’s guidelines. To confirm that the difference in different lanes is due to the biological effect of the transfected plasmids, rather than technical differences, equal amounts of lacz in each plate were transfected as a co-transfection plasmid to balance the difference in transfection efficiency.

### 2.2. Western Blot

After harvesting, the cells were lysed in ice-cold lysis buffer (50 mM Tris (pH 8.0), 150 mM NaCl, 1% Nonidet P-40, 1% sodium deoxycholate, 1% SDS, 1× protease inhibitor mixture (Roche, Basel, Switzerland)) for 30 min and centrifuged at 4 °C, 13,000 rpm for 10 min to remove insoluble material. The soluble protein concentration was determined by Bradford assay. Protein samples (60 μg) were separated by SDS-PAGE and transferred to nitrocellulose blotting membranes (Bio-Rad, Hercules, CA, USA). The membranes were treated with block buffer (5% non-fat milk in 0.1% TBST (20 mM Tris-HCl pH 8.0, 150 mM NaCl, 0.1% Tween-20)) at room temperature for 1 h. The membranes were then incubated with primary antibodies overnight at 4 °C, then washing (3 × 12 min) in PBS/Tween 20, followed by incubating with secondary antibodies in blocking buffer at room temperature for 2 h. Finally, washing (3 × 12 min) in PBS/Tween 20 again. The signals were detected and measured using LICOR Odyssey system (LI-COR, Lincoln, NE, USA). All the western blots were repeated at least three times.

### 2.3. Statistical Analysis

Data were analyzed using Prism 5.0 software (Graphpad Software, Inc., La Jolla, CA, USA). Results are presented as the mean ± standard deviation of three independent experiments. The difference between the means were tested by the One-way ANOVA testing or Student’s *t*-test, *p* < 0.05 was considered to indicate a statistically significant difference.

### 2.4. Prediction of SUMOylation Sites

In this study, prediction of the SUMOylation sites on the kinase domain on DAPK1 was performed using GPS-SUMO, which is a novel web server that can be used to predict potential SUMOylation sites (http://sumosp.biocuckoo.org/) [35]. 

### 2.5. Antibodies and Chemicals

Anti-GAPDH Antibody (2118) and anti-DAPK1 Antibody (3008) were purchased from Cell Signaling (Boston, MA, USA), anti-HA-Tag Antibody (902301) was purchased from Biolegend (San Diego, CA, USA), anti-flag Antibody (M20008) was purchased from Abmart (Shanghai, China), anti-SENP2 antibody (ab96865) was purchased from ABCAM (Cambridge, MA, USA), anti-Beta Galactosidase (β-gal) (*E. coli*) antibody (28449) was purchased from Rockland (Limerick, PA, USA). IRDye 800CW Goat-anti-Mouse (C60405-05), IRDye 680RD Goat-anti-Mouse (C60405-08), IRDye 800CW Goat-anti-Rabbit (C60607-15) and IRDye 680RD Goat-anti-Rabbit (C60329-15) were purchased from LI-COR (Lincoln, NE, USA).

MG132 was purchased from Calbiochem (LaJolla, NJ, USA) and used at 10 μM. Chloroquine and Cycloheximide (CHX) were purchased from Sigma (Louis, MO, USA) and used at 100 μM and 10 μg/mL, respectively. EST (E-64D) and Leupeptin were purchased from EMD Millipore Crop (Billerica, MA, USA) and used at 10 μg/mL and 200 μM respectively.

## 3. Results

**Figure 1:**
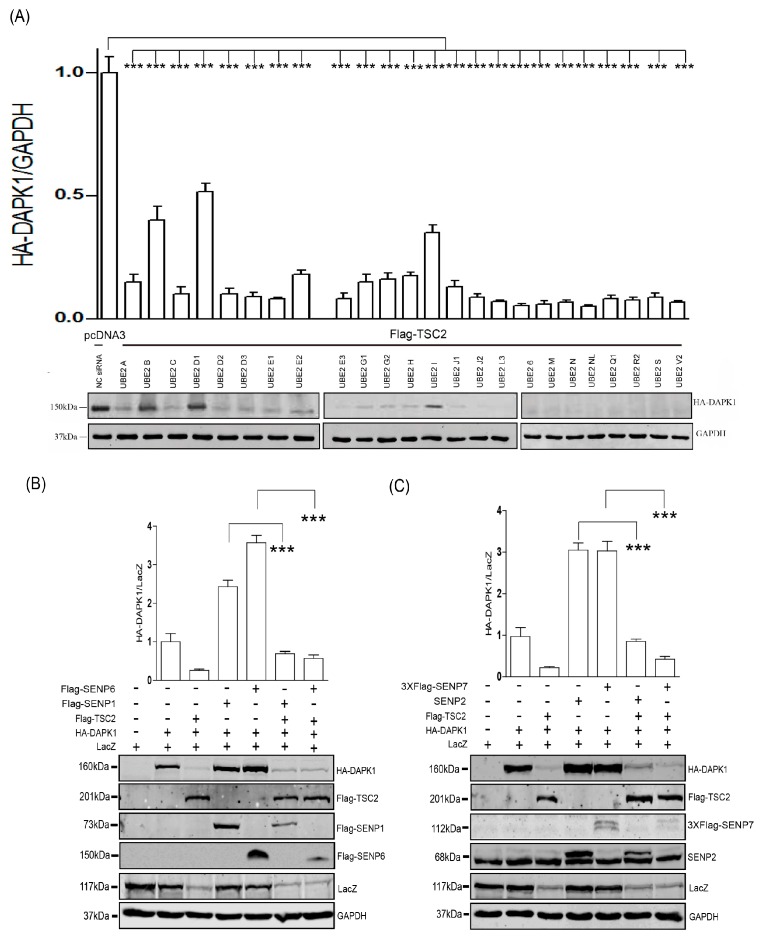
Inhibition of SUMO pathway partially restored DAPK1 protein levels at the presence of TSC2. (**A**) HEK293T was transfected with HA-DAPK1, Flag-TSC2, and different E2 siRNAs. (**B**,**C**) HEK293T cells were transfected with HA-DAPK1, LacZ, TSC2 and four different SENPs as indicated. Cell lysates were extracted 48 h post-transfection and immunoblotted with respective antibodies. The intensity of the bands was quantified using LICOR Odyssey software and represented by bar graphs. The experiments were repeated three times (n = 3), and representative images are presented. The representative western blot images are from different gels and each lane was loaded with the same amount of proteins. Data of triplicate assays are presented as mean ± S.D. * *p* < 0.05; ** *p* < 0.01; *** *p* < 0.001; NS, no significance. “+” indicated that the plasmid was transfected, “−” indicated that the plasmid was not transfected.

To search the potential ubiquitin or ubiquitin-like regulatory pathways involved in TSC2-mediated DAPK1 protein degradation, a screen using an E2 siRNA library was carried out. Co-transfection of the siRNAs targeting three E2s (UBE2B, UBE2D1 and UBE2I) up-regulated HA-DAPK1 protein levels upon co-transfection of TSC2 (Figure 1A). Of these three E2s, UBE2I, also named UBC9, is the E2 for SUMO, which has been shown to participate in protein degradation [36]. Therefore, we co-transfected four different SENPs with TSC2 and HA-DAPK1. Co-transfection of SENPs enhanced the level of HA-DAPK1 protein (Figure 1B,C), but not to the level without TSC2 co-transfection, suggesting inhibition of SUMO pathway is not able to abrogate the effect of TSC2 towards DAPK1 protein levels. 

**Figure 2:**
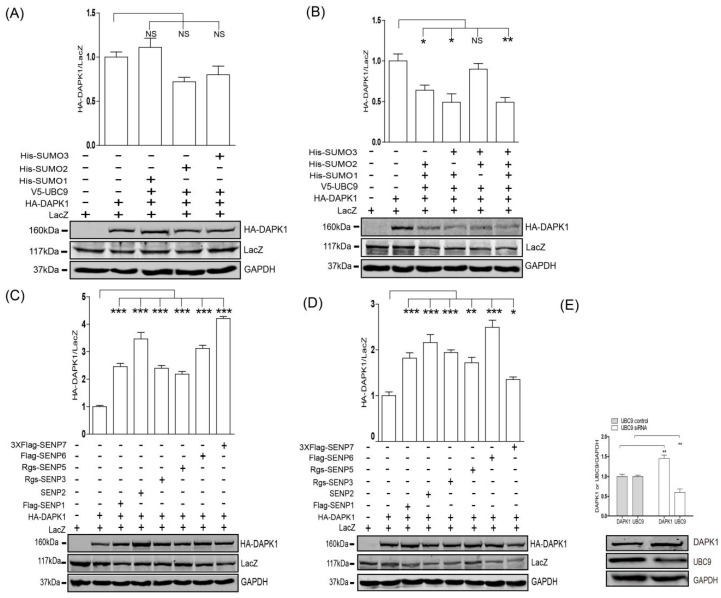
The SUMO pathway regulated the protein levels of DAPK1. HEK293T were transfected with (**A**) HA-DAPK1, LacZ, V5-UBC9, His-SUMO-1, His-SUMO-2 or His-SUMO-3, or (**B**) HA-DAPK1, LacZ, V5-UBC9, His-SUMO-1, His-SUMO-2 or His-SUMO-3, or (**C**) HA-DAPK1, LacZ and six different SENPs, or (**E**) control and UBC9 siRNA as indicated. HCT116 were transfected with (**D**) HA-DAPK1, LacZ and six different SENPs as indicated. Cell lysates were extracted 48 h post-transfection and immunoblotted with respective antibodies. The intensity of the bands was quantified using LICOR Odyssey software and represented by bar graphs. The experiments were repeated three times (n = 3) and representative images are presented. The representative western blot images are from different gels and each lane was loaded with the same amount of proteins. Data of triplicate assays are presented as mean ± S.D. * *p* < 0.05; ** *p* < 0.01; *** *p* < 0.001; NS, no significance. “+” indicated that the plasmid was transfected, “−” indicated that the plasmid was not transfected.

Next, we co-transfected individual SUMO construct with HA-DAPK1. Co-transfection of neither SUMO construct resulted in significant down-regulation of HA-DAPK1 protein levels (Figure 2A). However, when SUMO-1 was co-transfected with SUMO-2 or SUMO-3, it significantly stimulated the reduction of the HA-DAPK1 protein levels, whereas co-transfection of SUMO-2 and SUMO-3 did not seem to pose additive effect (Figure 2B). Furthermore, all six known SENPs significantly enhanced the levels of HA-DAPK1 protein when co-transfected with HA-DAPK1 in HEK293T cells (Figure 2C) and HCT-116 cells (Figure 2D). Moreover, consistent with the exogenous expression data, when endogenous UBC9 was knocked down using siRNA, the endogenous DAPK1 protein also significantly increased (Figure 2E), suggesting SUMO pathway is able to regulate DAPK1 protein levels without simultaneously manipulation of TSC2-related pathway.

**Figure 3:**
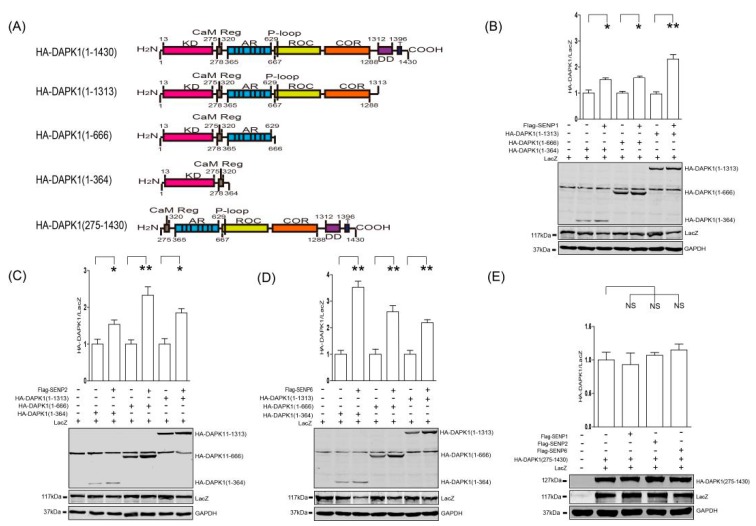
SENPs up-regulated DAPK1 protein levels via the 1-364 kinase domain. (**A**) A diagram illustrating the panel of DAPK1 deletion mutants. (**B–D**) The DAPK1 deletion mutants were co-transfected with LacZ and either Flag-SENP1 (**B**), Flag-SENP2 (**C**) or Flag-SENP6 (**D**), as indicated. (**E**) HA-DAPK (275–1430) mutant was co-transfected with LacZ and either Flag-SENP1, Flag-SENP2 or Flag-SENP6. Cell lysates were extracted 48 h post-transfection and immunoblotted with respective antibodies. The intensity of the bands was quantified using LICOR Odyssey software and represented by bar graphs. The experiments were repeated three times (n = 3) and representative images are presented. The representative western blot images are from different gels and each lane was loaded with the same amount of proteins. Data of triplicate assays are presented as mean ± S.D. * *p* < 0.05; ** *p* < 0.01; *** *p* < 0.001; NS, no significance. “+” indicated that the plasmid was transfected, “−” indicated that the plasmid was not transfected.

To further elucidate the underlying molecular mechanisms, a deletion series of DAPK1 constructs was created (Figure 3A) and co-transfected with three different SENPs. SENP1 (Figure 3B), SEP2 (Figure 3C) and SENP6 (Figure 3D) significantly enhanced the expression levels of all the deletion mutants, suggesting SUMO pathway regulates DAPK1 protein levels via the kinase domain. This is further confirmed when three SENPs displayed no effect towards the level of HA-DAPK1 (275–1430) lacking the kinase domain (Figure 3E).

**Figure 4:**
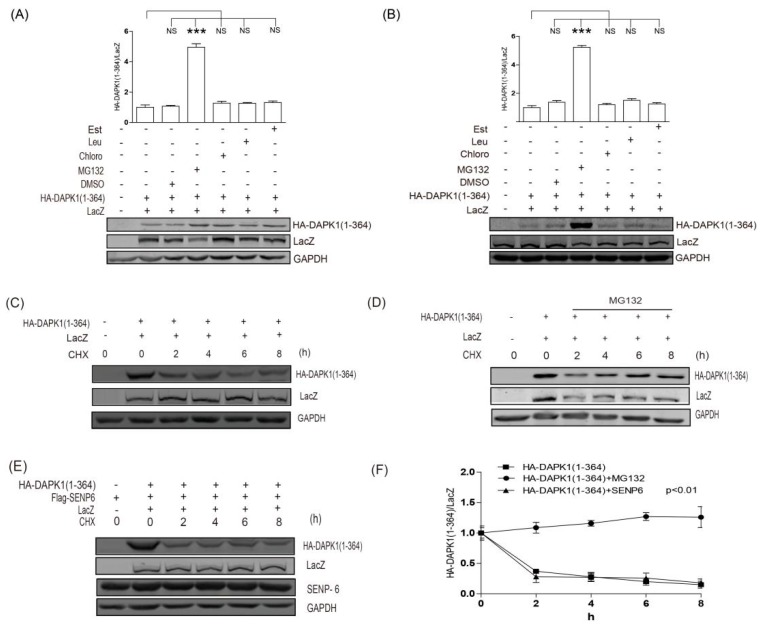
SUMO pathway did not regulate the protein degradation of HA-DAPK1 (1–364). (**A**) HEK293T cells or (**B**) HCT116 cells transfected with DAPK1 (1–364) and LacZ were exposed to MG132 (10 μM, 6 h) or leupeptin (200 μM), Est (10 μg/mL) and chloroquine (100 μM) for 24 h as indicated. (**C**) HEK293T transfected with LacZ, DAPK1(1–364) were exposed to 20 μg/mL CHX for 0–8 h as indicated. (**D**) HEK293T transfected with HA-DAPK1 (1–364) and LacZ were exposed to 10 μM MG132 and 20 μg/mL CHX for 0–8 h as indicated. (**E**) HEK293T transfected with LacZ, HA-DAPK1 (1–364) and Flag-SENP6 were exposed to 20 μg/mL CHX for 0–8 h as indicated. The statistical results of (**C**–**E**) are summarized in (**E**). Cell lysates were extracted and immunoblotted with respective antibodies. The intensity of the bands was quantified using LICOR Odyssey software and represented by trend lines. The experiments were repeated three times (n = 3) and representative images are presented. The representative western blot images are from different gels and each lane was loaded with the same amount of proteins. Data of triplicate assays are presented as mean ± S.D. * *p* <0.05; ** *p* < 0.01; *** *p* < 0.001; NS, no significance. “+” indicated that the plasmid was transfected, “−” indicated that the plasmid was not transfected.

Next, the HA-DAPK1 (1–364) was exposed to both proteasome and lysosome inhibitors. Only the proteasome inhibitor MG132 significantly enhanced the protein levels of HA-DAPK1 (1–364) in both HEK293T (Figure 4A) and HCT116 cells (Figure 4B), suggesting this kinase domain mutant HA-DAPK1 (1–364) is predominantly degraded via proteasome. In the protein stability assays using cycloheximide (CHX), MG132 significantly enhanced HA-DAPK1 (1–364) protein stability (Figure 4C,D,F), whereas co-transfection of SENP6 was unable to enhance the stability of HA-DAPK1 (1–364) protein (Figure 4C,E,F), indicating that the SUMO pathway does not regulate DAPK1 protein levels via protein degradation. This also suggested that the SUMO pathway was unlikely to control DAPK1 protein levels through direct conjugation.

**Figure 5:**
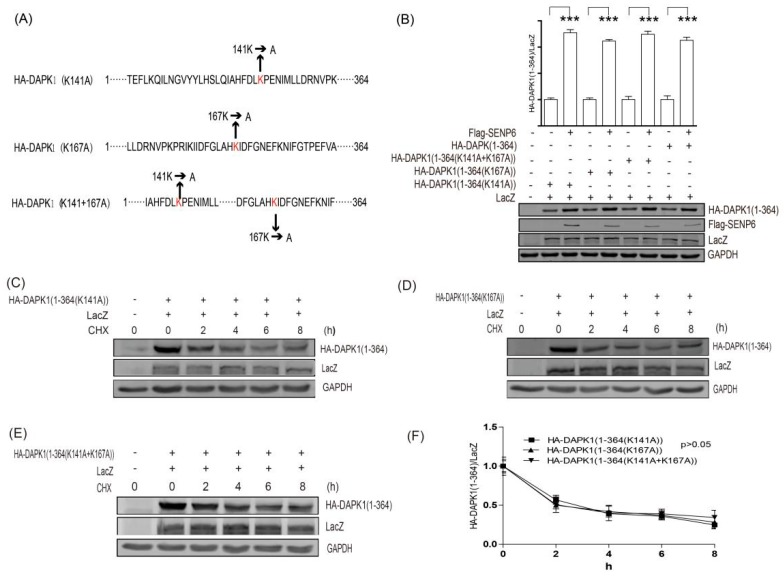
SUMO pathway did not regulate DAPK1 protein levels via direct conjugation. (**A**) A diagram illustrating the panel of DAPK1 (1–364) point mutants. (**B**) HEK293T cells were transfected with LacZ, SENP6 and the DAPK1 point mutants as indicated. (**C**–**E**) HEK293T transfected with LacZ, DAPK1 (1–364) point mutants were exposed to 20 μg/mL CHX for 0–8 h as indicated and the statistical results were summarized in (**F**). The intensity of the bands was quantified using LICOR Odyssey software and represented by bar graphs. The experiments were repeated three times (n = 3) and representative images are presented. The representative western blot images are from different gels and each lane was loaded with the same amount of proteins. Data of triplicate assays are presented as mean ± S.D. * *p* < 0.05; ** *p* < 0.01; *** *p* < 0.001; NS, no significance. “+” indicated that the plasmid was transfected, “−” indicated that the plasmid was not transfected.

Using the GPS-SUMO system, we identified two potential SUMO conjugation sites on HA-DAPK1 (1–364). Therefore, we mutated these two sites separately or simultaneously (Figure 5A). As expected, the mutation did not influence the effect of SENP6 on DAPK1 (1–364) (Figure 5B), supporting that the SUMO pathway does not regulate DAPK1 protein levels via direct conjugation. Next, we compared the protein stability of HA-DAPK1 (1–364) and the mutants with potential SUMO conjugation sties mutated. No mutants were able to enhance the protein stability of HA-DAPK1 (1–364) (Figure 5C–E).

## 4. Discussion

In our previous work, we discovered that TSC2 mediated the lysosomal degradation of DAPK1 via binding to the death domain of DAPK1 [20]. In this study, we discovered that inhibition of the SUMO pathway was able to enhance HA-DAPK1 protein levels at the presence of TSC2 co-transfection (Figure 1). However, the minimum domain of DAPK1 protein that the SUMO pathway is able to regulate is the kinase domain (Figure 3). The protein degradation of HA-DAPK1 (1–364) mutant is via proteasome (Figure 4). All these data suggest that the SUMO pathway regulates DAPK1 protein levels independent of TSC2. In this research, we co-transfected Lacz plasmid to balance the transfection variation and found that every single exogenous protein co-expressed with TSC2 shown decreased expression (Figure 1B,C). TSC2 is an important suppressor of mammalian target of rapamycin (mTORC1), which is a key regulator of translation and may be critical for exogenous protein levels. Therefore, when TSC2 was co-transfected, the expression of all other plasmids might be reduced due to inhibition of mTORC1 activity. The reason Glyceraldehyde-3-phosphate dehydrogenase (GAPDH) was stable could be because its translation is not mTORC1 dependent or its long half-life [37].

In two high-throughput mass spectrometry SUMOylation assays [38,39], DAPK1 protein was not found to be SUMOylated, suggesting DAPK1 may not be able to be SUMOylated, despite the presence of its potential SUMO conjugation sites. Our study is consistent with these two reports, but did not fully rule out the possibility that DAPK1 protein can be SUMOylated. It is only clear that even if DAPK1 protein can be SUMOylated, the SUMO conjugation is unlikely to affect the protein levels of DAPK1.

Although the SUMO pathway regulates DAPK1 protein levels, inhibition of the SUMO pathway does not change the protein stability of DAPK1 (Figure 4E). Therefore, the SUMO pathway probably regulates DAPK1 protein levels upstream of protein degradation. Considering that inhibition of the SUMO pathway enhanced the expression of exogenous HA-DAPK1, which only contains the coding region of DAPK1 mRNA, it is unlikely that SUMO pathway regulates DAPK1 protein levels through transcription. Recently, long noncoding RNAs (lncRNAs) have been shown to regulate gene expression at various levels, including chromatin modification, transcription and post-transcriptional processing [40]. Moreover, a new regulatory mechanism has been identified in which crosstalk between lncRNAs and mRNAs occurs by competing for shared microRNAs (miRNAs) response elements [41]. It is possible that the SUMO pathway regulates the expression of the miRNAs or lncRNAs targeting DAPK1, thus affect the levels of DAPK1 via these additional post-transcriptional regulatory pathways. It is also possible that the SUMO pathway regulates the expression levels or activity of proteins that are responsible for DAPK1 mRNA stability or translation.

In summary, our study discovered that the protein levels of DAPK1 can be regulated by the SUMO pathway. However, this regulation is not mediated via manipulation of DAPK1 protein degradation. The molecular mechanisms underlying this SUMO-mediated regulation of DAPK1 expression is still unclear. Further investigation is needed to elucidate this observation.

## References

[1] Bialik S., Kimchi A. (2014). The DAP-kinase interactome. Apoptosis.

[2] Wu B., Yao H., Wang S., Xu R. (2013). DAPK1 modulates a curcumin-induced G2/M arrest and apoptosis by regulating STAT3, NF-κB, and caspase-3 activation. Biochem. Biophys. Res. Commun..

[3] Yoo H.J., Byun H.J., Kim B.R., Lee K.H., Park S.Y., Rho S.B. (2012). DAPk1 inhibits NF-κB activation through TNF-α and INF-γ-induced apoptosis. Cell. Signal..

[4] Xie J.Y., Chen P.C., Zhang J.L., Gao Z.S., Neves H., Zhang S.D., Wen Q., Chen W.D., Kwok H.F., Lin Y. (2017). The prognostic significance of DAPK1 in bladder cancer. PLoS ONE.

[5] Niklinska W., Naumnik W., Sulewska A., Kozłowski M., Pankiewicz W., Milewski R. (2009). Prognostic significance of DAPK and RASSF1A promoter hypermethylation in non-small cell lung cancer (NSCLC). Folia Histochem. Cytobiol..

[6] Shiloh R., Bialik S., Kimchi A. (2014). The DAPK family: A structure–function analysis. Apoptosis.

[7] Huang Y., Chen L., Guo L., Hupp T.R., Lin Y. (2014). Evaluating DAPK as a therapeutic target. Apoptosis.

[8] Chou T.F., Chuang Y.T., Hsieh W.C., Chang P.Y., Liu H.Y., Mo S.T., Hsu T.S., Miaw S.C., Chen R.H., Kimchi A. (2016). Tumour suppressor death-associated protein kinase targets cytoplasmic HIF-1α for Th17 suppression. Nat. Commun..

[9] Chuang M., Hsiao T.I., Tong A., Xu S., Chisholm A.D. (2016). DAPK interacts with Patronin and the microtubule cytoskeleton in epidermal development and wound repair. Elife.

[10] Liu W.L., Yang H.C., Hsu C.S., Wang C.C., Wang T.S., Kao J.H., Chen D.S. (2016). Pegylated IFN-α suppresses hepatitis C virus by promoting the DAPK-mTOR pathway. Proc. Natl. Acad. Sci. USA.

[11] Benderska N., Schneider-Stock R. (2014). Transcription control of DAPK. Apoptosis.

[12] Raval A., Tanner S.M., Byrd J.C., Angerman E.B., Perko J.D., Chen S.S., Hackanson B., Grever M.R., Lucas D.M., Matkovic J.J. (2007). Downregulation of Death-Associated Protein Kinase 1 (DAPK1) in Chronic Lymphocytic Leukemia. Cell.

[13] Ivanovska J., Zlobec I., Forster S., Karamitopoulou E., Dawson H., Koelzer V.H., Agaimy A., Garreis F., Söder S., Laqua W. (2015). DAPK loss in colon cancer tumor buds: Implications for migration capacity of disseminating tumor cells. Oncotarget.

[14] Chan M.W., Chan L.W., Tang N.L., Tong J.H., Lo K.W., Lee T.L., Cheung H.Y., Wong W.S., Chan P.S., Lai F.M. (2002). Hypermethylation of multiple genes in tumor tissues and voided urine in urinary bladder cancer patients. Clin. Cancer Res..

[15] Gallagher P.J., Blue E.K. (2014). Post-translational regulation of the cellular levels of DAPK. Apoptosis.

[16] Jin Y., Blue E.K., Gallagher P.J. (2006). Control of death-associated protein kinase (DAPK) activity by phosphorylation and proteasomal degradation. J. Biol. Chem..

[17] Zhang L., Nephew K.P., Gallagher P.J. (2007). Regulation of death-associated protein kinase. Stabilization by HSP90 heterocomplexes. J. Biol. Chem..

[18] Lee Y.R., Yuan W.C., Ho H.C., Chen C.H., Shih H.M., Chen R.H. (2014). The Cullin 3 substrate adaptor KLHL20 mediates DAPK ubiquitination to control interferon responses. EMBO J..

[19] Laplante M., Sabatini D. (2012). mTOR Signaling in Growth Control and Disease. Cell.

[20] Yao L., Paul H., Susanne P., Jack S., Ted H., Craig S. (2011). Tuberous sclerosis-2 (TSC2) regulates the stability of death-associated protein kinase-1 (DAPK) through a lysosome-dependent degradation pathway. FEBS J..

[21] Lin Y., Stevens C., Harrison B., Pathuri S., Amin E., Hupp T.R. (2009). The alternative splice variant of DAPK-1, s-DAPK-1, induces proteasome-independent DAPK-1 destabilization. Mol. Cell. Biochem..

[22] Lin Y., Stevens C., Hupp T. (2007). Identification of a Dominant Negative Functional Domain on DAPK-1 That Degrades DAPK-1 Protein and Stimulates TNFR-1-mediated Apoptosis. J. Biol. Chem..

[23] Korolchuk V.I., Menzies F.M., Rubinsztein D.C. (2010). Mechanisms of cross-talk between the ubiquitin-proteasome and autophagy-lysosome systems. FEBS Lett..

[24] Huang C.J., Wu D., Khan F.A., Huo L.J. (2015). DeSUMOylation: An Important Therapeutic Target and Protein Regulatory Event. DNA Cell Biol..

[25] Kim K.I., Baek S.H. (2009). Chapter 7 Small Ubiquitin-Like Modifiers in Cellular Malignancy and Metastasis. Int. Rev. Cell Mol. Biol..

[26] Krumova P., Weishaupt J.H. (2013). Sumoylation in neurodegenerative diseases. Cell. Mol. Life Sci..

[27] Bohren K.M., Varsha N., Song J.H., Gabbay K.H., David O. (2004). A M55V polymorphism in a novel SUMO gene (SUMO-4) differentially activates heat shock transcription factors and is associated with susceptibility to type I diabetes mellitus. J. Biol. Chem..

[28] Liang Y.C., Lee C.C., Yao Y.L., Lai C.C., Schmitz M.L., Yang W.M. (2016). SUMO5, a Novel Poly-SUMO Isoform, Regulates PML Nuclear Bodies. Sci. Rep..

[29] Nan S.L., Marie-Claude G., Jaffray E.G., Hay R.T. (2009). Characterization of SENP7, a SUMO-2/3-specific isopeptidase. Biochem. J..

[30] Vertegaal A., Andersen J., Ogg S., Hay R., Mann M., Lamond A. (2006). Distinct and overlapping sets of SUMO-1 and SUMO-2 target proteins revealed by quantitative proteomics. Mol. Cell. Proteom..

[31] Bacco A.D., Ouyang J., Lee H., Catic A., Ploegh H., Gill G. (2006). The SUMO-specific protease SENP5 is required for cell division. Mol. Cell. Biol..

[32] Garone L., Fitton J.E., Steiner R.F. (2006). Characterization of a family of nucleolar SUMO-specific proteases with preference for SUMO-2 or SUMO-3. J. Biol. Chem..

[33] Chawon Y., Yonggang W., Debaditya M., Peter B., Nagamalleswari K., Alfred Y., Wilkinson K.D., Mary D. (2008). Nucleolar protein B23/nucleophosmin regulates the vertebrate SUMO pathway through SENP3 and SENP5 proteases. J. Cell Biol..

[34] Lima C.D., David R. (2008). Structure of the human SENP7 catalytic domain and poly-SUMO deconjugation activities for SENP6 and SENP7. J. Biol. Chem..

[35] Zhao Q., Xie Y., Zheng Y., Jiang S., Liu W., Mu W., Liu Z., Zhao Y., Xue Y., Ren J. (2014). GPS-SUMO: A tool for the prediction of sumoylation sites and SUMO-interaction motifs. Nucleic Acids Res..

[36] Tatham M.H., Geoffroy M.C., Shen L., Plechanovova A., Hattersley N., Jaffray E.G., Palvimo J.J., Hay R.T. (2008). RNF4 is a poly-SUMO-specific E3 ubiquitin ligase required for arsenic-induced PML degradation. Nat. Cell Biol..

[37] Franch H.A., Sooparb S., Du J., Brown N.S. (2001). A mechanism regulating proteolysis of specific proteins during renal tubular cell growth. J. Biol. Chem..

[38] Hendriks I.A., Vertegaal A.C. (2016). A comprehensive compilation of SUMO proteomics. Nat. Rev. Mol. Cell Biol..

[39] Hendriks I.A., Lyon D., Young C., Jensen L.J., Vertegaal A.C., Nielsen M.L. (2017). Site-specific mapping of the human SUMO proteome reveals co-modification with phosphorylation. Nat. Struct. Mol. Biol..

[40] Wilusz J.E., Hongjae S., Spector D.L. (2009). Long noncoding RNAs: Functional surprises from the RNA world. Genes Dev..

[41] Liu X.H., Sun M., Nie F.Q., Ge Y.B., Zhang E.B., Yin D.D., Kong R., Xia R., Lu K.H., Li J.H. (2014). Lnc RNA HOTAIR functions as a competing endogenous RNA to regulate HER2 expression by sponging miR-331-3p in gastric cancer. Mol. Cancer.

